# Changes in L-Carnitine Metabolism Affect the Gut Microbiome and Influence Sexual Behavior Through the Gut–Testis Axis

**DOI:** 10.3390/microorganisms13081751

**Published:** 2025-07-26

**Authors:** Polina Babenkova, Artem Gureev, Irina Sadovnikova, Inna Burakova, Yuliya Smirnova, Svetlana Pogorelova, Polina Morozova, Viktoria Gribovskaya, Dianna Adzhemian, Mikhail Syromyatnikov

**Affiliations:** 1 Department of Genetics, Cytology and Bioengineering, Voronezh State University, 394018 Voronezh, Russia; ms.babenkova@gmail.com (P.B.); gureev.bio.vsu@gmail.com (A.G.); ira-ivankina@yandex.ru (I.S.); vikagr04@gmail.com (V.G.); deanaajemyan@yandex.ru (D.A.); 2 Laboratory of Metagenomics and Food Biotechnology, Voronezh State University of Engineering Technology, 394036 Voronezh, Russia; vitkalovai@inbox.ru (I.B.); dyd16@mail.ru (Y.S.); zubkowa.sweta@gmail.com (S.P.); ms.cloud00.00@mail.ru (P.M.)

**Keywords:** L-carnitine, Mildronate, gut–seed axis, gut microbiome, male fertility

## Abstract

L-carnitine and Mildronate are substances that can significantly rearrange the energy metabolism of cells. This can potentially cause changes in the bacterial composition of the gut microbiome and affect testis functionality and male sexual health. Mice of the C57Bl/6 line were used. Sexual behavior was assessed using physiological tests, and gene expression patterns were assessed by qPCR. High-throughput sequencing of mouse fecal microbiota was performed. We showed that long-term administration of Mildronate has no significant effect on the intestinal microbiome, and there was a compensatory increase in the expression of genes involved in fatty acid and leptin metabolism. No impairment of sexual motivation in male mice was observed. Prolonged L-carnitine supplementation caused a decrease in alpha diversity of bacteria and a decrease in some groups of microorganisms that are components of a healthy gut microflora. A correlation was observed between the level of bacteria from Firmicutes phylum, indicators of sexual motivation of mice, and the dynamics of body weight gain. Our results may indicate that metabolic modulators can have a significant impact on the structure of the bacterial community of the gut microbiome, which may influence male sexual health through the gut–semen axis.

## 1. Introduction

One of the current topics is the study of the functionality of the testes and factors affecting their health and reproductive capacity in men. It is well known that the dietary supplement L-carnitine has a significant effect on fatty acid metabolism and energy processes in many organs and tissues. It promotes the transport of fatty acids across mitochondrial membranes, which, in turn, increases energy production in cells and improves oxidative metabolism. Studies show that L-carnitine may improve sperm quality and help increase sperm count [1]. Mildronate (3-(2,2,2-trimethylhydrazinium) propionate dihydrate; meldonium) is a widely known cytoprotective agent for cardiovascular diseases and a number of other pathologies of ischemic genesis, included in the pharmacopoeias of the CIS countries, Latvia, and Estonia. This drug was created at the Latvian Institute of Organic Synthesis by I. Ya. Kalvinsh and his colleagues. Historically, Mildronate was conceived by the authors as an aza-analogue of γ-butyrobetaine, and the goal was to create a drug for reversible inhibition of biosynthesis and reduction of carnitine concentration [2]. The Mildronate can inhibit the enzyme gamma-butyrobetaine hydroxylase, which causes a decrease in carnitine synthesis in the body. This, in turn, may limit the availability of L-carnitine for energy processes, especially under conditions of stress. In addition, Mildronate inhibits specific transporters, resulting in a significant decrease in tissue L-carnitine concentration, limiting fatty acid transport into mitochondria [3]. Nevertheless, Mildronate also promotes metabolic switch to more efficient glucose utilization, which may be advantageous in conditions of ischemia or increased exercise [4].

These compounds alter the flow of metabolic processes in the body, which can cause changes in the gut microbiome (GM) [5]. Considering that numerous studies on the gut–brain axis have proven the influence of the gut microbiota on brain function and activity [6] and that the hypothalamic–pituitary–testicular axis is considered as a classical neural regulator in the process of steroidogenesis [7], a similar influence on the testes can be assumed. The gut–testis axis is also thought to play an important role in the regulation of testis function, as the diversity of the microbiome is associated with different metabolic processes and immune responses, which, in turn, may affect their health. Thus, the GM acts as an important regulator that can have both positive and negative effects on reproductive function [8]. However, the relationship between testis functionality and the bacterial composition of the microbiota remains significantly less studied than the gut–brain axis.

The aim of this study was to investigate the effect of switching lipid metabolism with L-carnitine and Mildronate on sexual motivation and sexual behavior of mice, as well as the relationship of these parameters with the bacterial composition of the gut microbiome and the expression of metabolic genes in testes.

## 2. Materials and Methods

### 2.1. Object of Study

Two-month-old male and female mice of the C57Bl/6 line obtained from a branch of the Scientific Centre for Biomedical Technologies of the Stolbovaya Nursery (Moscow, Russia) were used in the experiment. The animals were kept under standard vivarium conditions at 25 °C, relative humidity of at least 40%, and a 12 h light/dark cycle, with free access to food and water.

### 2.2. Experiment Design

At the beginning of the experiment, 32 male mice were divided into three groups. The first group (*n* = 12) received clean drinking water ad libitum for four weeks, the second group (*n* = 10) received L-carnitine (KorolevPharm, Korolev, Russia) (200 mg/kg per day) together with drinking water, and the third group (*n* = 10) received Mildronate (Grindex, Riga, Latvia) (200 mg/kg per day) together with drinking water for four weeks. The experiment also included 17 female mice that received single injections of estradiol benzoate (50 µg in 100 µL of peanut butter) 72 h before a test to assess male sexual behavior and an injection of progesterone (400 µg in 200 µL of peanut butter) 3 h before this test. The behavior of males after 4 weeks was assessed using the Sexual Behavior Assessment Test and the Object Recognition Test. Fecal samples of male mice were collected for DNA extraction. Afterwards, the mice were killed. RNA samples from testes were isolated for analysis.

### 2.3. Test to Assess Sexual Behavior

The test is performed in the dark under dim red light for 60 min. Males and females were placed in different transparent containers for 30 min for acclimatization. The female was placed with the male, and the following parameters were evaluated: time to the first sniffing of the female by the male; time from placing the female to the first attempt of sexual intercourse; average time between successful introgressions; number of introgressions; and average duration of sexual intercourse.

### 2.4. Measurement of Gene Expression

Total RNA was isolated from testicular tissue using the commercial kit “ExtractRNA” (Eurogen, Moscow, Russia) according to the protocol. Reverse transcription was carried out using a set of reagents for cDNA production on RNA matrix “REVERTA-L” (FBUN Central Research Institute of Epidemiology of Rospotrebnadzor, Moscow, Russia) according to the protocol. The gene expression level was assessed by quantitative PCR analysis. The reaction mixture (20 µL volume) included 4 µL of qPCRmix-HS SYBR, 1 µL of forward and reverse primer mix (Table 1), 1 µL of cDNA, and 14 µL of mQ. Reaction conditions were as follows: total denaturation at 95 °C for 3 min; denaturation at the beginning of the cycle at 95 °C for 30 s; primer annealing at 59 °C for 30 s; and elongation at 72 °C for 30 s. The number of cycles was 45.

### 2.5. Extraction and Assessment of DNA Quality and Quantity

Total DNA was isolated from each obtained fecal sample using a commercial HiPure Microbiome DNA Kit (Magen, Guangzhou, China). Extraction was performed according to the manufacturer’s protocol. The amount of total DNA obtained was determined using a Nano-500 fluorimeter (Hangzhou Allsheng Instruments Co., Ltd., Hangzhou, China) and a commercial Equalbit 1x dsDNA HS Assay Kit (Vazyme, Nanjing, China). Purity and impurity were assessed using a Nano-500 spectrophotometer (Allsheng, Hangzhou, China) at wavelengths of 230, 260, and 280 nm. The quality of the obtained preparation was determined using electrophoresis in 2% agarose gel.

### 2.6. Library Preparation and Sequencing on the DNBSEQ-G50 Platform

For further sequencing of samples on the DNBSEQ-G50 platform (MGI, Shenzhen, China), libraries were prepared. First, fragmentation and ligation of adapters were performed using the commercial MGIEasy Fast FS Library Prep Module (MGI, China) and MGIEasy UDB Adapter Kit (MGI, China) according to the manufacturer’s protocol. Next, the PCR product yield was monitored using electrophoresis in 2% agarose gel. DNA concentration was assessed using a Nano-500 fluorimeter (Hangzhou Allsheng Instruments Co., Ltd., China) and a commercial Equalbit 1x dsDNA HS Assay Kit (Vazyme, China). Based on the obtained indicators, the PCR product mass was calculated. According to these calculations, several pools were formed. The concentration of each resulting pool was also measured. Then, denaturation was performed for further circularization of the formed single-stranded DNA using the commercial MGIEasy Dual Barcode Circularization Module kit (MGI, China). The quality of the resulting product was controlled by measuring the DNA concentration using a Nano-500 fluorimeter (Hangzhou Allsheng Instruments Co., Ltd., China) and a commercial QuDye ssDNA Assay Kit (Lumiprobe, Moscow, Russia). After that, the amount of µL of each pool that must be added when creating a superpool was calculated. Circularization efficiency was also calculated. Then, the concentration of the superpool was estimated, and the amount of µL of the superpool corresponding to 60 fmol was calculated. At the last stage, DNB was created using the commercial DNBSEQ-G50RS High-throughput Sequencing Kit FCL PE100/FCS PE150 (MGI, China). Next, the concentration was measured on a Nano-500 fluorimeter (Hangzhou Allsheng Instruments Co., Ltd., China) using a commercial QuDye ssDNA Assay Kit (Lumiprobe, Russia). Then, the sequencer was loaded using a DNBSEQ-G50RS Sequencing Flow Cell FCL (MGI, China) and a cartridge and reagents from the commercial DNBSEQ-G50RS High-throughput Sequencing Kit FCL PE100/FCS PE150 (MGI, China). All manipulations were performed according to the manufacturer’s protocols.

### 2.7. Bioinformatics and Statistical Analysis

Statistical analysis was performed using Statistica 12 software. The results were expressed as the mean value ± standard error of the mean. The statistical significance of differences between groups was assessed using the Kruskal–Wallis test. The correlation between the composition of the male mouse microbiome and physiological tests was assessed using the Spearman correlation coefficient (*p* < 0.01). Only statistically significant differences (*p* < 0.05) are discussed in this paper.

The quality of the raw metagenomic data was assessed using FastQC (FastQC v0.12.1) [9]. Technical sequences were trimmed using flexbar (flexbar version: 3.5.0) [10]. Human and host sequences from the samples were removed by comparing metagenomic reads against human (GCF_000001405.40) and mouse (GCF_000001635.27) reference genomes using the Bowtie2 tool [11]. Taxonomic profiling of samples was performed using MetaPhlAn 4 (Version 4.1.1) with standard bacterial, viral, and eukaryotic databases [12].

Statistical manipulations were performed in the R environment. Alpha diversity was assessed using the Shannon index, and the Bray–Curtis difference metric was used to analyze beta diversity. Differences in alpha diversity were assessed using the non-parametric Mann–Whitney test. The ADONIS function was used to assess differences in diversity between groups. Differential species abundance was analyzed with the MaAsLin2 package using a multivariate regression model. An adjusted *p*-value ≤ 0.05 was considered a statistically significant result. Results were presented as mean values ± standard deviations (SDs).

## 3. Results

### 3.1. Dynamics of Body Weight of Mice

**Figure 1:**
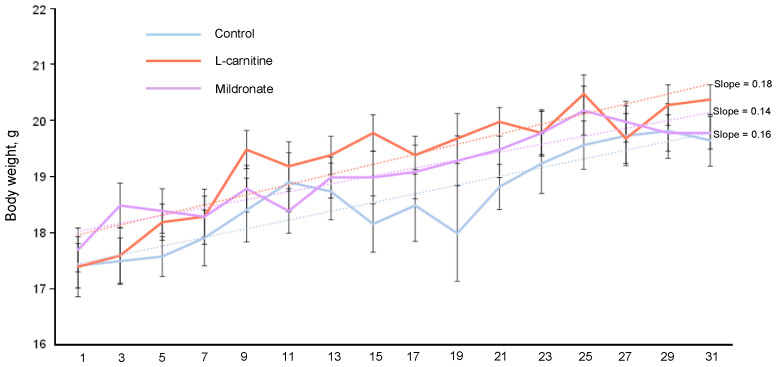
Dynamics of body weight of male mice.

As a result of systematic measurement of the body weights of mice of all studied groups, an increase in body weight was registered in the control group by 12.64%, in the group receiving L-carnitine by 17.24%, and in the group of mice receiving Mildronate, where body weight at the end of the experiment increased by 11.86%. The coefficient of dynamics of change in the weight of the mice, calculated as a linear approximation of the weight readings, was the maximum in the group of mice receiving Mildronate (0.18) and the minimum in the group of mice receiving L-carnitine (0.14) (Figure 1). At the same time, no statistically significant differences in the weight of mice on the last day of the experiment were observed.

### 3.2. Assessment of Gene Expression Levels

**Figure 2:**
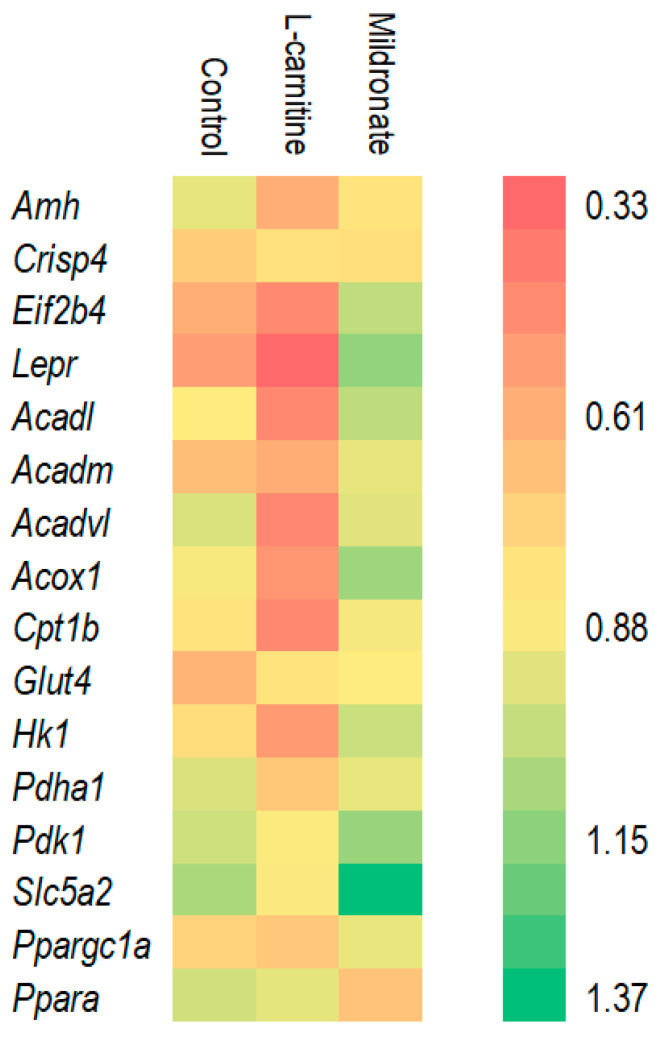
Relative gene expression levels in testes.

It was found that in the group of mice taking L-carnitine, there was a decrease in the expression of genes encoding various acetyl-CoA dehydrogenases (*Acadl*, *Acadm*, and *Acadvl*) in testes. On the contrary, the expression of these genes increased in the group of mice receiving Mildronate, which suggests the possibility of this drug modulating lipid metabolism. To maintain reproductive function, it is important to maintain physiological levels of leptin hormone [13]. The *Lepr* gene encodes the leptin receptor protein, and its expression level was increased 2.37-fold in the group of males taking Mildronate compared to the control (Figure 2). Thus, taking Mildronate did not impair the expression of genes affecting lipid and glucose metabolism and transport and also increased the expression of genes affecting spermatozoa formation.

### 3.3. Assessment of the Sexual Behavior of Male Mice

**Figure 3:**
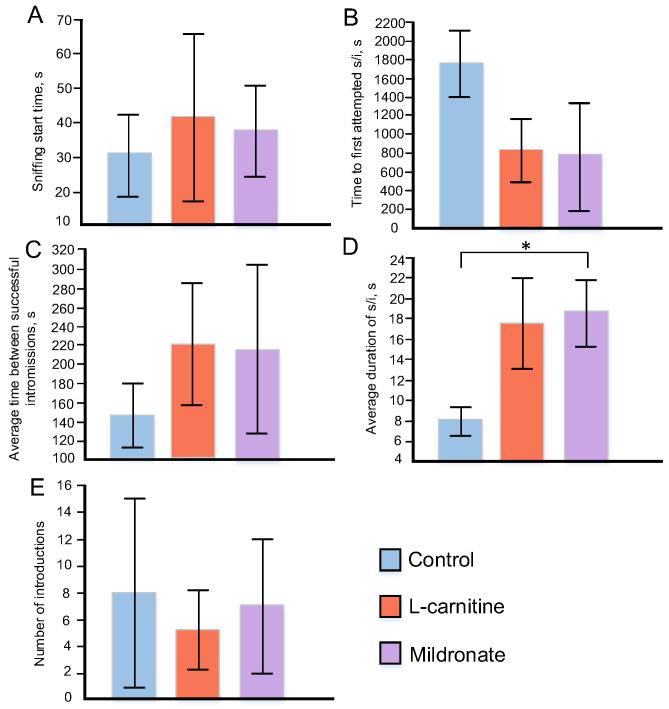
Variations in indices assessing the sexual behavior of male mice: time until the male first sniffs the female (**A**); time from planting the female to first attempted sexual intercourse (**B**); mean time between successful intromissions (**C**); mean duration of sexual intercourse (**D**); number of intromissions (**E**). * *p* ≤ 0.05.

In the group of mice receiving L-carnitine, the time to initiate the first attempt of sexual intercourse was reduced by 2.07 times and in the group receiving Mildronate by 2.31 times compared to the control, but the differences were not statistically significant. However, males receiving Mildronate had a higher mean sexual intercourse duration, 2.28 times higher compared to the controls (*p* = 0.012). In the group of mice receiving L-carnitine, this index was 2.11 times higher relative to the control. The time to the first sniffing of the female by the male in the group receiving L-carnitine increased by 38% and in the group receiving Mildronate by 24%. The time between attempts in the groups receiving L-carnitine and Mildronate increased by 68% and 62%, respectively, compared to the control, but the differences were not statistically significant (Figure 3).

### 3.4. Gut Microbiome Analysis

In total, 11 phyla, 104 classes, 108 orders, 126 families, 436 genera, and 564 bacterial species were identified. A total of 391 bacterial species were identified as previously unclassified, reflecting the fact that the mouse GM is a poorly understood consortium.

**Figure 4:**
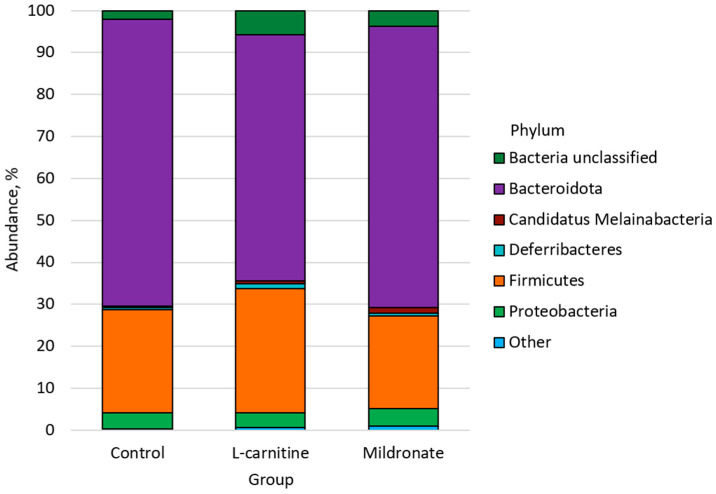
Types of bacteria found in the fecal microbiome of the mice studied.

The bacterial types found in the study groups are shown in Figure 4.

Bacteroidota was the most abundant phylum in all groups, with an abundance of 68.33% ± 6.01 in the control group, 58.58% ± 9.00 in the L-carnitine group, and 67.09% ± 4.80 in the Mildronate group. Firmicutes was the next most abundant phylum in the control (24.56% ± 5.35), L-carnitine (29.56% ± 6.33), and Mildronate (22.00% ± 3.71) groups. The third most abundant phylum was Proteobacteria in the control (3.73% ± 0.84) and Mildronate (4.22% ± 0.90) groups, being the fourth most abundant phylum in the L-carnitine group (3.55% ± 0.90). The next most abundant phylum in the L-carnitine group (5.75% ± 1.63) was Bacteria unclassified, whose abundance in the control group was 2.06% ± 0.54 and, in the Mildronate group, 3.72% ± 1.23. The abundance of Deferribacteres was 0.52% ± 0.26 in the control group, 1.07% ± 0.39 in the L-carnitine group, and 0.76% ± 0.39 in the Mildronate group. The abundance of the phylum Candidatus Melainabacteria was 1.25% ± 0.34 in the Mildronate group, compared with the control (0.42% ± 0.17) and the L-carnitine group (0.81% ± 0.47). Candidatus Saccharibacteria was predominant in the Mildronate group (0.33% ± 0.10), while its abundance in the control and L-carnitine groups was 0.23% ± 0.08 and 0.28% ± 0.10, respectively. Verrucomicrobia was also more abundant in the Mildronate group (0.39% ± 0.19) than in the control group (0.02% ± 0.01) and the L-carnitine group (0.15% ± 0.11). The next most common phylum in the L-carnitine group (0.19% ± 0.15) was Tenericutes, whose abundance in the control group was 0.10% ± 0.03 and, in the Mildronate group, 0.09% ± 0.04. In the Mildronate group (0.15% ± 0.10), the Spirochaetes type predominates, while the content of this type in the control and L-carnitine groups is 0.02% ± 0.01 and 0.06% ± 0.03, respectively. Actinobacteria was present only in the control group (0.02% ± 0.01).

**Figure 5:**
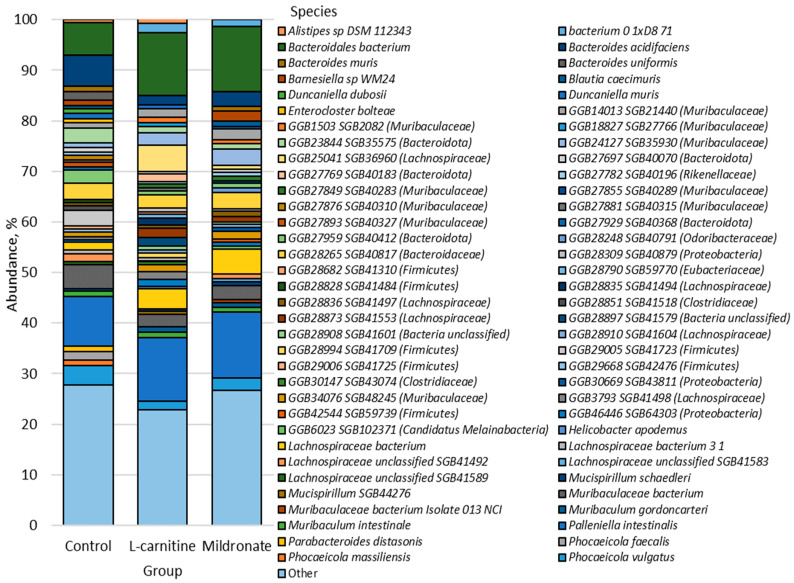
Bacterial species found in the microbiome of the mice studied.

The prevalence of 68 species was greater than 0.5%; they were the most numerous species in the studied groups; all other species were grouped as “Other” (Figure 5).

The most numerous bacterial species in the studied groups was *Palleniella intestinalis*, whose content in the control group was 9.85% ± 2.59, in the L-carnitine group 12.56% ± 2.15, and Mildronate 13.11% ± 2.54. There was also a significant abundance of the *Bacteroidales* bacterium species in the L-carnitine (12.46% ± 2.82) and Mildronate (12.85% ± 3.09) groups, as well as in the control group 6.44% ± 2.26. The next most common was *Muribaculaceae* bacterium in the control (4.66% ± 0.86), L-carnitine (2.48% ± 0.55), and Mildronate (2.79% ± 0.44) groups. Also, the abundance of *GGB28265 SGB40817 (Bacteroidaceae)* was 3.12% ± 1.10 in the control group, 2.55% ± 1.11 in the L-carnitine group, and 3.17% ± 0.54 in the Mildronate group. The number of bacteria of the species *Phocaeicola vulgatus* was the most numerous in the control (3.83% ± 1.52) and Mildronate groups (2.38% ± 0.66). The *Lachnospiraceae* bacterium was most common in the L-carnitine (3.87% ± 0.01) and Mildronate (4.87% ± 0.03) groups. The next most abundant species in the L-carnitine group was *GGB25041 SGB36960 (Lachnospiraceae)*, with a content of 5.15% ± 1.52. *Bacteroides acidifaciens* was one of the predominant species in the control group microbiome (6.20% ± 1.98).

**Figure 6:**
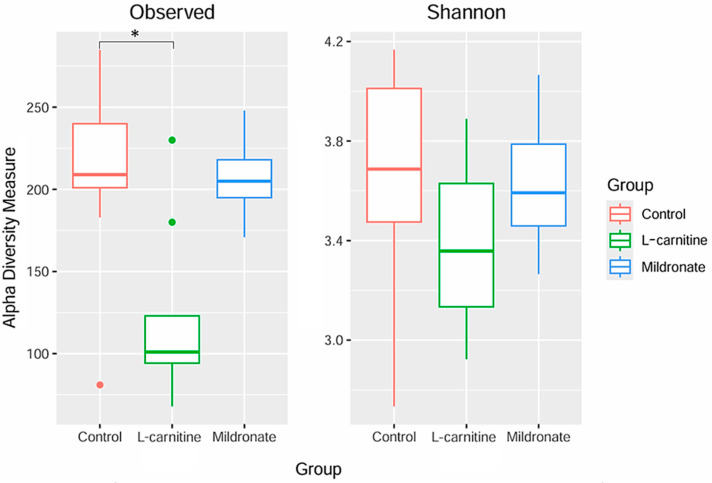
Alpha diversity of the microbiome of the studied groups. * *p* ≤ 0.05.

Alpha diversity analysis was also performed using the observed species diversity measures and the Shannon index (Figure 6). The alpha diversity of the fecal microbiome of mice can be characterized as high, since the Shannon index value was greater than three for all studied groups (Table 2). Statistically significant differences were found only in the observed species diversity measure between the control and L-carnitine groups (204 ± 20.80 vs. 119 ± 15.97, *p* = 0.012) (Figure 6).

**Figure 7:**
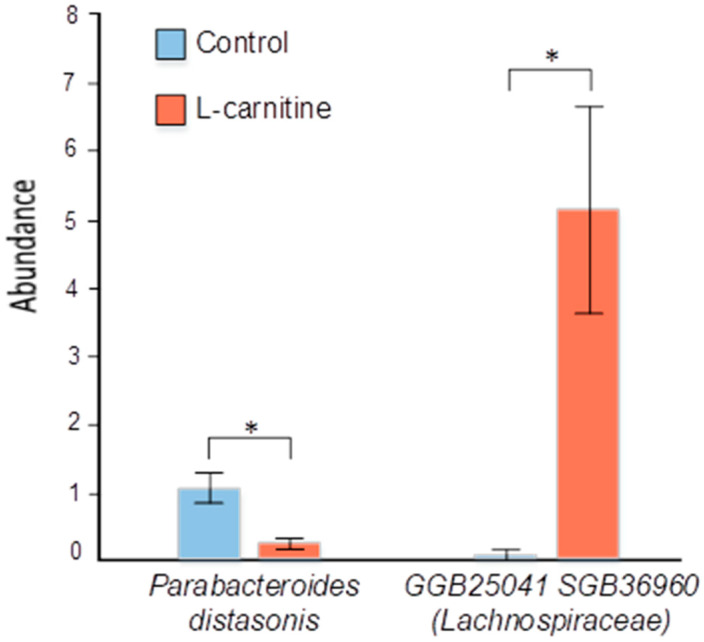
Differences in fecal microbiome composition between the control group and the L-carnitine group. * *p* ≤ 0.05.

**Figure 8:**
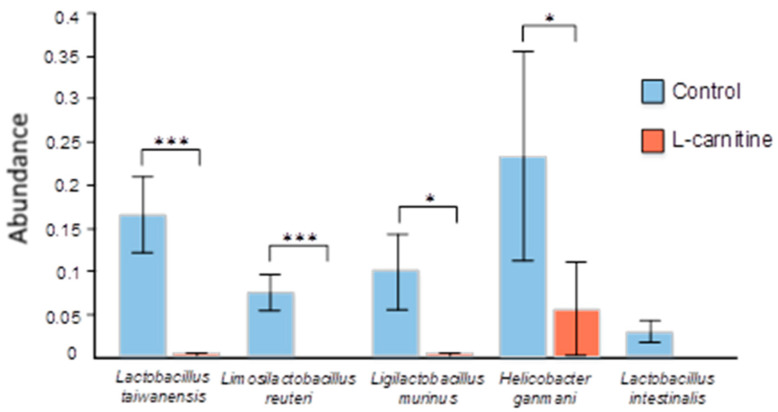
Differences in fecal microbiome composition between the control group and the L-carnitine group. * *p* ≤ 0.05; *** *p* ≤ 0.001.

Differential abundance analysis revealed statistically significant differences at the species level between the control group and the L-carnitine group (Figure 7 and Figure 8). Thus, in the L-carnitine group, compared with the control group, we observed a decrease in the number of the species *Lactobacillus taiwanensis* (0.165% ± 0.044 vs. 0.002% ± 0.002, *p* = 6.10 × 10^−7^), *Limosilactobacillus reuteri* (0.075% ± 0.021 vs. 0, *p* = 3.27 × 10^−4^), *Parabacteroides distasonis* (1.052% ± 0.222 vs. 0.208% ± 0.070, *p* = 1.42 × 10^−2^), *Ligilactobacillus murinus* (0.099% ± 0.044 vs. 0.002% ± 0.002, *p* = 2.44 × 10^−2^), *Helicobacter ganmani* (0.233% ± 0.121 vs. 0.056% ± 0.054, *p* = 3.12 × 10^−2^), and *Lactobacillus intestinalis* (0.029% ± 0.013 vs. 0, *p* = 4.29 × 10^−2^). In contrast, in the L-carnitine group compared to the control group, we observed an increase in the amount of the species GGB25041 SGB36960 (*Lachnospiraceae*) (5.151% ± 1.523 vs. 0.081% ± 0.078, *p* = 4.42 × 10^−2^).

In the fecal microbiome of mice in the Mildronate group, compared to the control group, there was an abundance of GGB45513 SGB63185 (*Oscillospiraceae*) (0.093% ± 0.023 vs. 0.006% ± 0.003, *p* = 0.033) and GGB27860 SGB40294 (*Muribaculaceae*) (0.208% ± 0.034 vs. 0.064% ± 0.026, *p* = 0.043). At the same time, a decrease in the content of bacteria of the species *Lactobacillus taiwanensis* was revealed in the Mildronate group compared to the control group (0.165% ± 0.044 vs. 0.010% ± 0.005, *p* = 2.62 × 10^−6^), as well as *Limosilactobacillus reuteri* (0.075% ± 0.021 vs. 0.004% ± 0.003, *p* = 0.001), *Helicobacter ganmani* (0.233% ± 0.121 vs. 0.002% ± 0.002, *p* = 0.006), and *Lactobacillus intestinalis* (0.029% ± 0.013 vs. 0, *p* = 0.043) (Figure 8).

### 3.5. Evaluation of Correlations Between Bacterial Content and Physiological Indices of Male Mice

Significant (*p* < 0.01) correlations were found between the indices of sexual motivation of mice and the content of some groups of bacteria in the intestinal microbiome. The abundance of Tenericutes representatives was negatively correlated with the time between sexual attempts (r_s_ = −0.49, *p* < 0.01). Also, the level of some groups of the Firmicutes type correlated with the time of onset of the first sexual attempt (r_s_ = 0.54, *p* < 0.01) and negatively correlated with the duration of sexual intercourse (r_s_ = −0.51, *p* < 0.01) and the number of intromissions (r_s_ = −0.5, *p* < 0.01). In the L-carnitine group, there was a trend towards an increase in the coefficient of body weight change. It was found that this coefficient in males correlated with the number of Firmicutes (r_s_ = 0.48, *p* < 0.01), and this index was negatively correlated with the abundance of *Bacteroides uniformis* (r_s_ = −0.58, *p* < 0.01). Also, the coefficient of mice weight dynamics correlated with the difference between sexual attempts (r_s_ = 0.55, *p* < 0.01).

## 4. Discussion

### 4.1. Gene Expression Changes

It was previously shown that inhibition of L-carnitine synthesis by Mildronate can affect metabolism at the transcriptomic level. It was shown that short-term Mildronate treatment caused a compensatory increase in the expression of *LPL*, *CPT-Iα* and *β*, *mtGPAT*, *DGAT*, *CTPpct*, and *apoB* genes in the heart [4,14]. Long-term Mildronate treatment caused a compensatory increase in *Acox1* expression in the liver and brain and *Acadl* expression in the liver [15]. Increased expression of *cpt1* and *ehhadh* was observed in the liver of *Danio rerio* that received Mildronate [16]. In this experiment, we observed an average twofold increase in the expression of genes encoding various acetyl-Coa dehydrogenases as well as acetyl-Coa oxidase in the testes of mice treated with Mildronate compared to those mice that received L-carnitine, which may indicate that compensatory effects from inhibition of L-carnitine synthesis and reabsorption have an effect on gene expression not only in the heart, liver, and brain but also in the testes. At the same time, we observed a twofold increase in expression of the glucose transporter *Slc5a2* but not *Glut4* in the testes, which may indirectly indicate that the metabolic switch from fatty acid oxidation to glucose oxidation is observed in testes similarly to other organs. It was previously shown that short- and long-term Mildronate treatments promote an increase in the expression of genes encoding these transporters in the heart [4,15].

We also observed a Mildronate-induced threefold increase in the expression of the *Lepr* gene, which encodes the leptin receptor. This may also indirectly indicate metabolic rearrangement, since leptin is hypothesized to function as a negative feedback signal in the regulation of energy balance [17]. For example, mice with obesity show decreased expression of *Lepr* in the testes, which was accompanied by decreased fertility [18]. In our experiment, minimal *Lepr* expression was observed in the group of mice receiving L-carnitine. No signs of obesity were observed in these mice, but it is worth noting the tendency to increase body weight in mice receiving L-carnitine, where we also found a correlation with weight gain.

### 4.2. Сhanges in Sexual Behavior

There is no reason to believe that metabolic switching had any negative effect on the sexual motivation of the mice. The only parameter that was statistically significantly higher in Mildronate-treated mice was copulatory rate, which may reflect a mixture of sexual motivation and potency, but this parameter is of little informative value in the absence of other significant changes [19]. It is likely that nitric oxide (NO) stimulation may be involved in the increase in copulatory rate, as well as the tendency to decrease the time to the first attempt at sexual intercourse in mice treated with Mildronate. Mildronate is known to increase nitric oxide synthase (NOS) activity and enhance NO production [19,20], which is known to be essential for maintaining penile erectile function [21].

Despite the generally recognized fact that L-carnitine can improve male sexual function at the indicated concentrations of 100, 200, and 350 mg/kg/day [22,23,24,25,26], there are reports that chronically oral administration of L-carnitine induces testicular injury, which was manifested by an increase in the number of abnormal spermatozoa and a decrease in their motility [27]. Although we did not observe in our study L-carnitine-induced impairment of sexual behavior in mice, we observed some signs in the disturbance of the bacterial composition of the intestinal microbiome. There is increasing evidence for the existence of a microbiota–gut–testis axis, where changes in the composition of the gut microbiota through various complex mechanisms affect male reproductive function [28].

### 4.3. Mechanism of Action of the Gut–Testis Axis

GM dysbiosis can lead to the development of systemic inflammation and triggering of the immune response, and the GM is also involved in the antioxidant defense of the body, which is very important for testicular function, since spermatozoa are particularly sensitive to oxidative stress [29]. But the influence of the gut microbiota does not end with the modulation of these processes: the GM affects fertility through its own metabolites such as LPS and vitamin K and those synthesized from dietary sources, including short-chain fatty acids (SCFA), polyunsaturated fatty acids, and amino acid derivatives, as well as metabolites initially synthesized by the host and subsequently modified by the GM, such as secondary bile acids and hydroxysteroid dehydrogenase. SCFAs are involved in the regulation of sperm production and motility [30]. In addition, they enhance the activity of glutathione peroxidase (GPx) and superoxide dismutase (SOD) enzymes in 45-week-old adult roosters, promoting testosterone secretion and testicular growth [31]. SCFAs can improve gut microbiota by regulating lipid metabolism to improve spermatogenesis and enhance sperm volume and fertility through the production of n-3 polyunsaturated fatty acids [32].

One of the key mechanisms of action of SCFAs is their interaction with specific receptors such as GPR41 (FFAR3) and GPR43 (FFAR2). These receptors are expressed in various tissues [33,34]. In addition, SCFAs inhibit pro-inflammatory pathways, which reduces inflammation and protects the testicles from damage [35]. An important aspect is also the effect of SCFAs on the intestinal barrier. Butyrate strengthens the tight contacts between enterocytes, preventing bacterial toxins from entering the systemic bloodstream [36]. This reduces the level of chronic inflammation, which can negatively affect testicular function by suppressing testosterone synthesis and impairing sperm quality. Another important mechanism is epigenetic regulation. Butyrate, being a histone deacetylase inhibitor, alters histone acetylation, which leads to increased expression of genes that may also be important for steroidogenesis [33,37]. For example, it is able to increases the activity of the *StAR* gene (steroidogenic acute regulatory protein), which ensures the transport of cholesterol into the mitochondria, the first and key stage of testosterone synthesis. SCFAs also enhance the expression of the enzymes CYP11A1 and CYP17A1, which are involved in steroidogenesis [38,39]. The metabolic effects of SCFAs also play an important role. Acetate serves as a substrate for the synthesis of cholesterol, which is a precursor to all steroid hormones, including testosterone [34].

Another important metabolite modified by microbiota is tryptophan. It is metabolized into indole, and then in the gut, the GM further metabolizes indole into various derivatives such as indole-3-propionic acid (IPA) and 3-hydroxyindole, which significantly affect host health [40]. IPA inhibits GM dysbiosis and intestinal endotoxin leakage [41]. Indole-derived metabolites enhance testosterone secretion and increase StAR protein expression to attenuate cisplatin-induced testicular injury (II), inhibit OS, and inflammation [42]. Importantly, the GM is also involved in the conversion of L-carnitine and other compounds to trimethylamine (TMA), which subsequently forms pro-inflammatory trimethylamine N-oxide (TMAO) in the liver. TMAO, in turn, can lead to epigenetic modifications as well as the formation of N-nitrous, which damage cellular DNA [43]. The GM may affect sperm quality and offspring health through effects on host gene expression and epigenetic modifications. This effect is manifested in DNA methylation patterns or regulation of histone modifications [30].

In the last decade, there has also been increasing evidence suggesting that androgens can significantly remodel the gut microbiota through complex pathways. In turn, the GM is also involved in the regulation of androgen production and metabolic processes. The GM has also been reported to have an effect on testosterone levels, as after transplantation of microbiota from adult male mice to immature female mice, the latter had increased testosterone levels. The GM has also been discovered as one of the main pathways of androgen metabolism. Glucuronidated androgens can be excreted into the small intestine through bile [8].

### 4.4. The Impact of Changing Bacterial Strains

The results of our study showed that the most common bacterial types in all three study groups were *Bacteroidota*, *Firmicutes*, and *Proteobacteria*.

*Bacteroidota* and *Firmicutes* are known to be among the predominant bacterial types in the intestine [44]. However, an increased ratio of *Firmicutes* to *Bacteroidota* in the body is usually associated with poor health [45] and other metabolic disorders [46]. The *Bacteroidota* type is known to produce mainly acetate and propionate, while *Firmicutes* produce more butyrate [47]. In turn, butyrate is believed to be able to have health-promoting effects [48]. It is known that the presence of propionate in the colon results in the release of glucagon like peptide-1 (GLP-1) and peptide YY (PYY) by L-enteroendocrine cells, which, in turn, affects appetite [49]. The presence of acetate in the body increases fat accumulation and appetite, which can lead to obesity [48]. Moreover, the *Firmicutes* type has a huge number of bacteria known for their ability to produce short-chain fatty acids [50].

#### 4.4.1. Firmicutes

Statistically significant differences were found for the bacterial species *GGB25041 SGB36960* of the family *Lachnospiraceae*. Members of the *Lachnospiraceae* family are known to be capable of fermenting non-digestible dietary carbohydrates to produce metabolites, including SCFAs, and participate in nutrient acquisition and energy homeostasis [51]. SCFAs may affect spermatogenesis [30]. Also, the abundance of this family increased during subtherapeutic antibiotic therapy at an early age in mice with obesity [52].

In addition, representatives of *Lachnospiraceae* are potential sources of antimicrobial and immunomodulatory compounds. Thus this family is capable of developing intestinal therapeutics by its properties [53]. However, the role of the family *Lachnospiraceae* is controversial. The increased number of representatives of several genera in this family may have both positive and negative effects on several diseases: obesity, diabetes, IHD, and depressive syndrome [54]. The results of our study showed an increase in the species *GGB25041 SGB36960* of the family *Lachnospiraceae* in the L-carnitine group relative to the control group, but the functions of the identified species are unknown, as no literature data describing *GGB25041 SGB36960* were found.

A decrease in *Lactobacillus* species was recorded in the L-carnitine and Mildronate groups, indicative of intestinal dysbiosis and a negative consequence of L-carnitine administration. *Lactobacillus taiwanensis* as well as *Lactobacillus intestinalis*, like many lactobacilli, have probiotic properties, maintain the balance of the microbiota [55], and produce lactic acid. Their reduction can lead to the deterioration of gut health and increased susceptibility to disease [56]. Studies also show that this group is useful for improving sperm parameters, increasing testosterone levels and reducing lipid peroxidation in the testicles [57,58].

The reduction of *Limosilactobacillus reuteri* bacteria in the L-carnitine group is also a negative consequence of diet, as this species has an antimicrobial effect expressed in the production of reuterin, a broad-spectrum antibiotic agent [59]. *L. reuteri* is also found in breast milk and healthy intestinal microbiota [60], is used as prevention of intestinal infections, and is more effective compared to other probiotics, as demonstrated in comparative studies [61]; moreover, *Limosilactobacillus reuteri* has the potential to inhibit *Helocobacter pylori* infection [62,63]. Sperm quality parameters, such as total and progressive motility, acrosome integrity, and other kinematic parameters, were significantly improved after administration of *Limosilactobacillus reuteri* in dogs [57]. This group of microorganisms enhances mitochondrial activity in sperm through specific factors such as AMPK and SIRT1 and increases the activity of proteins associated with mitochondrial function in sperm, which increases the motility of mouse spermatozoa [64].

*Ligilactobacillus murinus* was reduced in the L-carnitine group compared to the control. It is a common probiotic. Studies using *L. murinus* prevented intestinal ischemia/reperfusion-induced intestinal injury while improving survival rates in mice [65]; also, *L. murinus* is a quantitative biomarker of intestinal health [66] *Lactobacillus* is identified as a Gram-positive bacterium and may be associated with the synthesis of SCFA. Excessive abundance of *Lactobacillus* in men can alter the pH of seminal fluid and cause an abnormal microenvironment of spermatogenesis. Dysbiosis of these key microbiota can have a significant impact on progressive sperm motility [67]. Our data also reflected a dual picture of the effect of *Firmicutes* representatives on the sexual function of mice, where there was an inverse correlation with the duration of sexual intercourse and the number of intromissions, but there was also a correlative direct dependence of this type with the time of the first attempt of sexual intercourse, which can be interpreted as a negative effect on the sexual behavior of males.

An increase in the *Oscillospiraceae* family was observed in the Mildronate group, which is a positive response to the use of Mildronate, as a decrease in this organism plays a role in the pathogenesis of immune-mediated inflammation and progression of inflammatory bowel disease [68]. There are studies supporting the relationship between the reduction of bacteria of the *Oscillospiraceae* family and the progression of ulcerative colitis and the decreased gene expression of bacterial enzymes activated by fatty acids [69]. It is also worth noting that *Oscillospiraceae* or *Ruminococcaceae* contain representatives capable of generating secondary fatty acids [70]. Their effects may be mediated through hormonal changes, toxic effects, and systemic metabolic disorders [71].

#### 4.4.2. Bacteroidota

Intestinal bacteria of the *Bacteroidota* type are able to produce enzymes aimed at digesting complex food sugars, possibly contributing to the host energy intake [72]. In addition, calorie restrictive diets and weight loss lead to an increase in the type *Bacteroidota* in the intestine, thereby leading to a decrease in fat mass [48]. Some previous animal studies have shown that an abundance of *Bacteroides* and *Prevotella* is negatively correlated with sperm motility [73]. It was found that the predominance of *Prevotella* genus corresponded to poor semen quality in men with obesity [74]. The *Prevotellaceae* family is partly responsible for the transformation of TMA-derived compounds, but more importantly, it produces SCFAs [43].

It has previously been shown that treatment with *Parabacteroides distasonis* can protect mice from insulin resistance, as well as enhance intestinal integrity and reduce systemic inflammation in mice [75]. Also, recent studies have revealed that the *P. distasonis* bacterium is able to protect the body against multiple sclerosis, type II diabetes, colorectal cancer, and inflammatory bowel disease [76]. In addition *P. distasonis* is able to exert a protective effect not only in acute but also in chronic models of colitis by increasing the frequency of innate type 3 lymphoid cells in the colon, in addition to improving the integrity of the intestinal epithelium [77]. Our study showed a decrease in the abundance of this bacterium in the L-carnitine group relative to the control group of the study. Possibly, such indicators may indicate an unfavorable effect of this modulator on the intestinal integrity of mice, which is consistent with the literature data. *P. distasonis*, a spermine-advantaged strain that has been found to be reduced in testicular dysfunction, is able to restore testicular function by increasing polyamine levels [78]. The functions of spermine include antioxidant protection, inhibition of lipid synthesis, regulation of ion channels, and maintenance of normal reproductive physiology [79].

The increase in the *Muribaculaceae* family is a positive outcome of Mildronate administration, as they produce short-chain fatty acids and also show cross-reactivity with probiotics such as *Bifidobacterium* and *Lactobacillus* [80]. The family *Muribaculaceae* is attached to the mucosal layer and is a symbiotic user of myxoglycan in the gut, showing a strong correlation with inflammatory bowel diseases [81]. The family also has a negative correlation with obesity [82] and diabetes mellitus [83]. *Muribaculaceae* restores sperm concentration and testosterone levels by regulating ornithine levels. Ornithine is associated with the activity of the low-density lipoprotein receptor gene, which is indirectly associated with testosterone synthesis [84].

#### 4.4.3. Proteobacteria

It has been observed that high abundance of *Proteobacteria* type in the GI tract of mice has been associated with dysbiosis. The association of this type with inflammation and metabolic syndrome has also been reported [85]. In turn, it has been reported that the predominance of this type of bacteria in the body is noted in the intestine of newborn mice. In turn, the absence of B cells in adult mice is able to alter the ratio of the intestinal microbiota, suggesting a link between these cells and the abundance of *Proteobacteria* [86]. In addition, it has been shown that IgA-deficient mice exhibited persistent intestinal colonization of *Proteobacteria*, which, in turn, led to permanent intestinal inflammation and increased susceptibility to neonatal and adult models of intestinal damage [87,88]. Due to this, it can be inferred that increased abundance of *Proteobacteria* may act as a potential microbial marker of disease occurrence [89]. The relative abundance of *Proteobacteria*, which may lead to intestinal inflammation and tumors, was significantly higher in men with asthenozoospermia [65]. The genus of bacteria *Asticcacaulis* of the type of *Proteobacteria* has a negative correlation with the number and motility of spermatozoa in the study of the effects of thermal stress on the testes. This group also showed a negative correlation with L-arginine and a positive correlation with retinol, and the metabolism of these molecules is disrupted in infertility models [90].

The reduction of *Helicobacter ganmani* in mice fed with L-carnitine by 4-fold relative to the control and Mildronate by 116-fold relative to control may be considered as a positive effect, since *H. ganmani* has been associated with various gastrointestinal diseases [91]. In the study, duodenitis as well as chronic gastritis with severe infiltration was observed in all mice co-colonized with *H. ganmani* [92]. Gastritis was characterized by severe infiltration of polymorphonuclear leukocytes in the basal part of the intrinsic lamina, the submucosa of the corpus callosum, and in the adjacent muscle layers. However, there are also works that indicate unclear pathogenic activity of *H. ganmani* [93]. Bacteria from *Gammaproteobacteria* such as *Escherichia coli*, *Citrobacter*, *Klebsiella pneumoniae,* and *Shigella* and *Achromobacter* from the *Betaproteobacteria* strain have enzymes such as CntA, CntB, YeaW, and YeaX that encode a gene that can convert all food compounds, including choline, betaine, gamma-butyrobetaine, and L-carnitine, into TMA [43].

#### 4.4.4. Deferribacteres

*Deferribacteres* is a new type registered in 2001 [94]. The bacterial type *Deferribacteres* in the intestinal flora plays a role in iron metabolism and iron balance in the GI tract [95]. *Deferribacteres* have also been shown to be increased in mice with food allergies [96]. Moreover, several studies have shown that increased *Deferribacteres* abundance plays a pathogenic role in the development of colitis induced by sodium dextran sulfate [97]. However, *Mucispirillum* strains, also of the *Deferribacteres* type, were positively correlated with testosterone and sperm activity [98]. In our study, the presence of this bacterial type was demonstrated in all the studied groups. However, greater abundance was in the L-carnitine group, which may indicate the possibility of any metabolic conditions associated with increased abundance of *Deferribacteres*.

#### 4.4.5. Tenericutes

*Tenericutes* are completely inverted and are mainly distributed in the esophagus and stomach [99]. Tenericutes are known to usually act as commensals or parasites of humans, animals, insects, and even plants [100]. The relative abundance of *Tenericutes* was found to be correlated with butyrate synthesis pathways [101]. However, members of this type were associated with lower Body Mass Index (BMI) and triglycerides and higher High-Density Lipoprotein (HDL) levels and were strongly associated with increased acetate and short-chain fatty acids [102]. *Tenericutes* were also negatively correlated with levels of sex hormones and significantly positively correlated with indicators of reproductive damage such as NO and NOS [103]. The negative impact of this group of representatives of the intestinal microbiome on the male sexual system is also confirmed by our data on the correlation with the value of time between attempts of sexual intercourse.

Patients with asthenozoospermia had lower richness and diversity (α-diversity and β-diversity) of intestinal flora [65]. On this basis, in the L-carnitine group, reduced α-diversity may also affect testes and sexual behavior. Reducing the amount of *Tenericutes* can have a beneficial effect on the body by altering bile acid metabolism and reducing inflammation [104].

#### 4.4.6. Other

At the end of our study, an increase was observed in the abundance of bacterial types—*Candidatus Melainabacteria*; *Candidatus Saccharibacteria*; *Verrucomicrobia*; *Spirochaetes*—in the experimental groups. Currently, there is a lack in the number of studies aimed at investigating the role of *Candidatus Melainabacteria* in the intestinal microbiota. However, it has been shown that the abundance of this type in the small intestine of mice was also reduced in mice treated with Simotang oral fluid relative to mice treated with cisplatin [105]. It is known that *Candidatus Saccharibacteria* can be associated with all markers of obesity, including BMI, weight, fat mass, muscle mass, waist circumference, and lipid accumulation [106]. It was noted that infertility models with insulin resistance showed higher levels of the *Saccharibacteria* phylum and lower levels of the *Actinomycetota* and *Verrucomicrobia* phyla compared with the control group without this symptom [107].

It was found that the bacterial type *Verrucomicrobia* is a beneficial bacterium ubiquitous in the healthy human gut, capable of controlling the inflammation process, and a decrease in the abundance of this type may indicate microbial community instability or gut dysbiosis [108].

The results of previous studies have shown that Zika Virus infection is able to increase the abundance of bacteria belonging to the *Spirochaetes* type compared to uninfected mice. In turn, this infection is capable of affecting gut bacterial composition and colon tissue homeostasis in adult immunocompetent mice [109]. However, the *Spirochaetes* type is known to demonstrate increased sensitivity to antimicrobials [110]. Our data demonstrate the predominance of *Spirochaetes* in the Mildronate group of the study relative to the control and L-carnitine groups.

## 5. Conclusions

Thus, metabolic regulators L-carnitine and Mildronate have a significant effect on the structure of the bacterial community of the intestinal microbiota. The most significant changes were observed in the group of mice receiving L-carnitine. We did not observe significant deviations in the sexual behavior of male mice, although correlations were observed between L-carnitine intake, increased body weight, and a tendency to worsen some behavioral parameters, which may indicate that longer-term use of the supplement could potentially have a negative effect on male sexual health. Mildronate had an effect on male sexual behavior by increasing the time of sexual intercourse, but this parameter did not have a separate negative effect on fertility and could potentially be related to increased NO production. Long-term use of Mildronate was not associated with any deterioration in the bacterial microbiome and or sexual behavior of mice. In general, we can conclude that the gut–testis axis represents one of the least explored areas in microbiome research, with the number of publications addressing gut–testis interactions being nearly two orders of magnitude lower than those investigating the gut–brain axis (70 results vs. 9971 in PubMed (access date 24 June 2025)). This striking disparity persists despite growing evidence that microbial metabolites can directly influence testicular function through multiple pathways, including immune modulation, endocrine disruption, and oxidative stress regulation. Our study reveals several critical knowledge gaps that warrant urgent attention: the lack of mechanistic understanding regarding how specific bacterial taxa influence spermatogenesis, the absence of established dose–response relationships for microbial metabolites affecting male reproduction, and the paucity of human studies controlling for confounding factors like diet and comorbidities. Our data, while contributing important preliminary data on microbiome–testis interactions, underscore the need for more comprehensive mechanistic studies to fully elucidate gut–testis axis.

## Figures and Tables

**Table 1:** Primer sequences used for expression assessment.

№	Gene Name	Primer Sequence
1	*Gapdh*	F:5′-CATCACTGCCACCCAGAAGACTG-3′ R: 5′-ATGCCAGTGAGCTTCCCGTTCAG-3′
2	*Eif2b4*	F: 5′-GCTTGCAACAGGTAGCTTGT-3′ R: 5′-CCCCTCACTCACCTTGACAT-3′
3	*Crisp4*	F:5′-ATGGATGTGGGTATGGCAGT-3′ R: 5′-GCAGCTGAACTCCAACTCAC-3′
4	*Lepr*	F: 5′-CTTTCCTGTGGACAGAACCAGC-3′ R: 5′-AGCACTGAGTGACTCCACAGCA-3′
5	*Amh*	F:5′-CCGCTATTTGGTGCTAACCGTG-3′ R: 5′-AAGGCTTGCAGCTGATCGATGC-3′
6	*Acadl*	F:5′-CCATGGCAAAATACTGGGCA-3′ R: 5′-TTGGTACCACCGTAGATCGG-3′
7	*Acadm*	F:5′-AGGGTTTAGTTTTGAGTTGACGG-3′ R: 5′- CCCCGCTTTTGTCATATTCCG-3′
8	*Acadvl*	F:5′-CTACTGTGCTTCAGGGACAAC-3′ R: 5′-CAAAGGACTTCGATTCTGCCC-3′
9	*Acox1*	F:5′-TAACTTCCTCACTCGAAGCCA-3′ R: 5′-AGTTCCATGACCCATCTCTGCC-3′
10	*Cpt1b*	F:5′-AGGCACTTCTCAGCATGGTC-3′ R: 5′-CATCTCGAACATCCACCCGT-3′
11	*Glut4*	F:5′-CCTCCCGCCCTTAGTTG-3′ R: 5′-CTGCAAAGCGTAGGTACCA-3′
12	*Hk1*	F:5′-GTTCGAGAAGATGGTGAGCG-3′ R: 5′-AGAGTTCCCATCCCGTTTCA-3′
13	*Pdha1*	F:5′-GTTTTGGGCGTGGCTTCG-3′ R: 5′-GGCTTGCCGGCTTCTG-3′
14	*Pdk1*	F:5′-TCCTGGACTTCGGGTCAGT-3′ R: 5′-GTATGCTGAGCTCCAGGCCAA-3′
15	*Ppargc1a*	F:5′-ATGTGTCGCCTTCTTGCTCT-3′ R: 5′-CACGACCTGTGTCGAGAAAA-3′
16	*Ppara*	F:5′-AGAGCCCCATCTGTCCTCTC-3′ R: 5′-ACTGGTAGTCTGCAAAACCAAA-3′
17	*Slc5a2*	F:5′-TGGTGTTGGCTTGTGGTCTA-3′ R: 5′-ATGTTGCTGGCGAACAGAGA-3′

**Table 2:** Number of fecal bacteria and measures of their diversity.

Group	Observed Species	Shannon Index
Control	204	3.64
L-carnitine	119	3.39
Mildronate	212	3.63

## Data Availability

Metagenome data are available in the NCBI BioProject database (BioProject ID: PRJNA1234534).

## Abbreviations

The following abbreviations are used in this manuscript:

UDB	Union Database
cDNA	Complementary DNA
PCR	Polymerase chain reaction
dsDNA	Double-stranded DNA
ssDNA	Single-stranded DNA
PE	Paired end
FCL	Flow cell large
FCS	Flow cell small
MetaPhlAn	Metagenomic phylogenetic analysis
MaAsLin	Multivariate analysis by linear models
NO	Nitric oxide
NOS	Nitric oxide synthase
GI	Gastrointestinal
PYY	Peptide YY
GLP-1	Glucagon like peptide-1
BMI	Body mass index
HDL	High-density lipoprotein
SCFAs	Short-chain fatty acids
GPx	Glutathione peroxidase
SOD	Superoxide dismutase
StAR	Steroidogenic acute regulatory protein
